# Evaluation of resting traps to examine the behaviour and ecology of mosquito vectors in an area of rapidly changing land use in Sabah, Malaysian Borneo

**DOI:** 10.1186/s13071-018-2926-1

**Published:** 2018-06-14

**Authors:** Rebecca Brown, Chua Tock Hing, Kimberly Fornace, Heather M. Ferguson

**Affiliations:** 1 Institute of Biodiversity, Animal Health and Comparative Medicine, College of Medical, Veterinary and Life Sciences, University of Glasgow, Glasgow, G12 8QQ UK; 2 Department of Pathobiology and Medical Diagnostics, Faculty of Medicine and Health Sciences, Universiti Malaysia Sabah, 88400 Kota Kinabalu, Sabah Malaysia; 3 Faculty of Infectious and Tropical Diseases, London School of Hygiene and Tropical Medicine, London, WC1E 7HT UK

**Keywords:** Resting behaviour, Sticky traps, Exophily, Zoonosis, Blood meal identification

## Abstract

**Background:**

Widespread deforestation occurring in the tropics is hypothesized to impact the transmission of vector-borne diseases (VBD). Predicting how environmental changes will impact VBD transmission is dependent on understanding the ecology and behaviour of potential vector species outside of domestic settings. However there are few reliable sampling tools for measuring the habitat preference and host choice of mosquito vectors; with almost none suitable for sampling recently blood-fed, resting mosquitoes. This study evaluated the use of two mosquito traps: the resting bucket (RB) and sticky resting bucket (SRB) traps relative to CDC backpack aspiration (CDC) for sampling mosquitoes resting in a range of habitats representing a gradient of deforestation. Eight habitats were selected for sampling around two villages in Kudat District, Malaysian Borneo, to reflect the range of habitats available to mosquitoes in and around human dwellings, and nearby forest habitats where reservoir hosts are present: secondary forest (edge, interior and canopy); plantations (palm and rubber); and human settlements (inside, under and around houses).

**Results:**

Over 31 days, 2243 mosquitoes were collected in 5748 discrete collections. Nine mosquito genera were sampled with *Aedes* and *Culex* species being present in all habitats and most abundant. RB and CDC backpack aspiration were most efficient for sampling *Culex* whereas CDC backpack aspiration and SRB were most efficient for *Aedes*. Most *Aedes* identified to species level were *Ae. albopictus* (91%), with their abundance being highest in forest edge habitats. In contrast, *Culex* were most abundant under houses. Most blood-fed mosquitoes (76%) were found in human settlements; with humans and chickens being the only blood source.

**Conclusions:**

RB and SRB traps proved capable of sampling mosquitoes resting in all sampled habitats. However, sampling efficiency was generally low (*c.*0.1 per trap per day), necessitating traps to be deployed in high numbers for mosquito detection. None of the traps were effective for sampling zoonotic malaria vectors; however, SRB collected relatively higher numbers of the dengue vector *Ae. albopictus*. The higher abundance of mosquitoes in forest edge habitats indicates the potential value of these traps for investigating sylvatic dengue transmission. This study has demonstrated the merits in application of simple resting traps for characterising mosquito vector resting behaviour outside of the home.

**Electronic supplementary material:**

The online version of this article (10.1186/s13071-018-2926-1) contains supplementary material, which is available to authorized users.

## Background

Vector-borne diseases are responsible for 17% of all infectious diseases contracted worldwide, impacting the public health and economic growth of primarily developing countries [1]. Vital to the control of vector-borne disease (VBDs) is an understanding of the ecology and behaviour of species responsible for pathogen transmission [2]. This is particularly crucial for tackling emerging VBDs where data on vector biology are scarce. One such example is the emergence of the primate malaria causative agent *Plasmodium knowlesi* in human populations in Southeast (SE) Asia over the past decade, with an epicentre in the State of Sabah in Malaysian Borneo [3, 4]. *Plasmodium knowlesi* is a simian malaria parasite whose primary hosts are long-tailed and pig-tailed macaques, and leaf-monkeys [5]. Human infection with *P. knowlesi* was previously thought to be rare [6]; however, the number of human infections reported in SE Asia has substantially increased in recent years [4, 7]. *Plasmodium knowlesi* now accounts for the largest proportion of malaria cases in people in Malaysian Borneo [3]. Other mosquito-borne diseases are present in this area including human malaria (*P. falciparum*, *P. vivax*, *P. malariae* [3]), filariasis [8–14], Japanese encephalitis [15], dengue [16–22], and chikungunya [23]. Cases of Zika were also recently reported [24]. Development of integrated vector control approaches with capacity to target this suite of mosquito VBDs would be of benefit in Malaysia and the numerous other settings where they co-occur.

The emergence of *P. knowlesi* in Sabah has been associated with rapid changes in land use [25]. From 1980 to 2010, the area of land covered by forest in Sabah decreased from 60% to 51% [26]. This change is largely attributable to conversion of forest to plantation to meet the increasing demand for palm oil [26]. Changes in land-use for agriculture have been associated with outbreaks of mosquito VBDs in other settings [27–29]. Proposed mechanisms for these increases include changes in soil conditions and drainage following deforestation that alter the availability of aquatic habitats for mosquito larvae [29–31]. Ground and water temperatures are higher in cleared than in forested areas [32, 33] which can speed up mosquito larval development and reduce the length of the adult gonotrophic cycle. Both these changes are expected to increase mosquito fitness and abundance [32–34]. Higher temperatures can also increase the rate of pathogen development in mosquitoes (e.g. malaria parasite development [33–35] and dengue virus [36]). Additionally, following forest removal, humans often migrate to new, cleared areas leading to an increase in frequency of contact between human and animal hosts [37]. Consequently deforestation has potential to increase a range of mosquito VBDs of public health importance [30]. This occurred in the Peruvian Amazon where *Anopheles* biting rates increased in deforested areas causing an upsurge in malaria cases [38] and also in Sarawak, Malaysia, where development of a palm oil plantation led to a reduction in malaria vectors but an increase in vectors of dengue virus [29].

The increase in *P. knowlesi* poses a significant challenge because the mosquito vector species responsible for transmission are unlikely to be targeted by conventional control strategies. For example, the primary vector of *P. knowlesi* in Sabah is *Anopheles balabacensis* [39]; a species that bites almost exclusively outdoors (exophilic) and has a relatively high survival rate [40]. Additionally, this vector species feeds extensively on the non-human primates that act as a reservoir for *P. knowlesi*. The two common methods of vector control in Malaysia, insecticide-treated nets and indoor residual spraying [41, 42], only provide protection from mosquitoes attempting to feed on people inside houses; and are thus unlikely to have much impact against exophilic and zoophilic species like *An. balabacensis*. These challenges are not unique to *P. knowlesi*. Several of the mosquito species responsible for other VBDs in the area are also exophilic and/or become infected from an animal reservoir. For example, Borneo experiences a sylvatic dengue transmission cycle between macaques and silver langurs [43], driven by forest *Aedes* species [44]. Currently evidence suggests that sylvatic dengue transmission is restricted to forests; however, several spillover cases into the human population have occurred [45, 46]. *Aedes niveus* is expected to be responsible for transmission in the forests of Sarawak and spillover to humans is driven by the exophilic *Ae. albopictus*, acting as a bridge vector, spanning a wider range of habitats including villages, agricultural areas and forests [46]. However, information about key vectors transmitting sylvatic dengue in Sabah is unknown. The human dengue serotypes spread by *Ae. aegypti* and *Ae. albopictus* in urban areas are believed to have originated from sylvatic dengue strains [45] and although currently sylvatic strains seem to be largely restricted to the forest, evidence suggests that these viruses do not require any adaptation time to replicate efficiently in humans [45]. This highlights the potential for epidemics to arise and stresses the need for reliable tools that can be used across a range of habitat types to characterise *Aedes* mosquito ecology and host preference to understand sylvatic dengue transmission in Sabah. Furthermore, both Japanese encephalitis (pigs, horses and donkeys [47]) and filariasis (e.g. cats, dogs and leaf monkeys [12, 48]) can be spread to humans from an animal reservoir. The control of this group of VBDs is clearly dependent on the development of novel vector control tools which can target vectors in multiple habitat types outside of the home [49].

The development of such control strategies is impeded by a lack of appropriate sampling tools for investigation of mosquito vector ecology outside of homes. Characterization of mosquito feeding behaviour and habitat use requires tools that sample both the host-seeking and resting population. However, most standard sampling methods can only be applied indoors. For example, host-seeking mosquitoes are frequently sampled using CDC light traps indoors (malaria vectors) [50–52] or BG sentinel traps (dengue vectors) [53–55]. Similarly resting mosquitoes are usually targeted by aspiration of mosquitoes from the inside of house walls (e.g. *Aedes* [56–58] and *Anopheles* [59]) or pyrethrum spray catch indoors [60]. Whilst host-baited traps have shown some success for sampling mosquitoes host-seeking on animals and humans outdoors [61–66], there are few methods for sampling mosquitoes resting in forest or other non-domestic habitats. Sampling resting mosquitoes is particularly vital for characterizing mosquito host choice. This is inferred by analysis of the blood meal of recently fed females to identify host preference. There are several methods for sampling mosquitoes resting in and around the home [59, 67–70] but these often give biased estimates of host choice by favouring humans and peridomestic animals [71, 72]. These techniques are rarely used to sample mosquitoes in wilderness areas away from homes. As yet, resting collections have largely been used to investigate diseases transmitted around the home, not ones that could be transmitted in forested habitats or that have a wild animal reservoir host. Recent work in Africa has evaluated standardized, portable and low-cost resting traps for collecting resting *Anopheles* in peridomestic settings [59, 70]. These have yet to be trialled for sampling mosquitoes resting in forest and other non-domestic habitats. Further to defining habitat use and host choice of vectors, there is a need for standardised resting collection techniques to monitor and detect alterations in mosquito behaviour. Changes to the environment and use of control methods can drive adaptations and shift patterns of behaviour in vector populations. An example of this is the use of insecticide-treated bed nets in Tanzania and Papua New Guinea which resulted in shifts to outdoor biting, time of biting and changes in host feeding behaviour [73, 74]. Land-use changes such as deforestation for cultivating palm oil also induce changes in mosquito behaviour [29, 38]; however, in order to detect shifts in host choice or resting behaviour, new methods are required that can span all available habitats, such as those arising from deforestation, to detect any differences occurring between them.

The aim of this study was to evaluate two new trapping methods for sampling mosquitoes resting within domestic, peridomestic, agricultural and forest settings in an area of Malaysian Borneo where multiple VBDs are present. Whilst the study encompassed investigation of the mosquito community in general, our focus was on known vectors of malaria, dengue and filariasis. We tested a simple bucket trap [59] and sticky trap [70] that were originally developed for sampling outdoor resting malaria vectors in Africa. These methods were compared with collections made using a CDC backpack aspirator. This is a standard method for sampling vectors resting inside houses [46, 68] and is occasionally used to collect insects resting on vegetation [75]. These techniques were compared across eight different habitat types representing a gradient of deforestation, with the aim of characterising the resting habitat preferences and host choice of potential mosquito vectors. This information will highlight the suitability of these novel techniques for understanding mosquito behaviour and ecology.

## Methods

### Study site selection

**Fig. 1:**
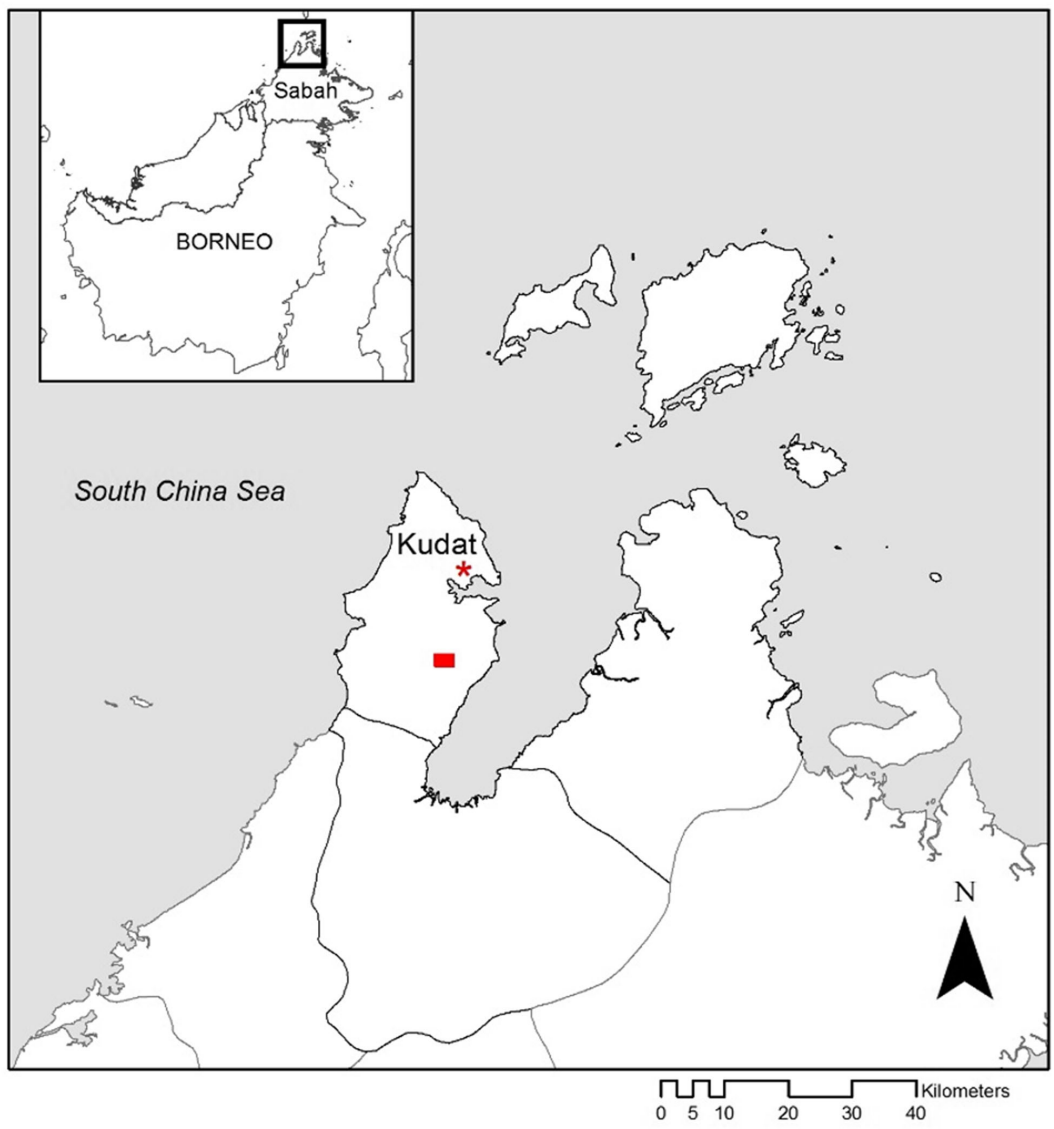
Map of Sabah in Malaysian Borneo with red rectangle in Kudat District indicating site used for investigating resting mosquito behaviour. The rectangle represents a 2 × 3 km grid intensively studied for macaque and mosquito ecology specifically in relation to *P. knowlesi* emergence

This study was conducted in the Kudat District of Sabah State in Malaysian Borneo (Fig. 1). Kudat was the focus of a successful community engaged and intersectoral approach to control *P. falciparum* malaria from 1987 to 1991 [76]. In recent years however, this district has experienced a high burden of human *P. knowlesi* cases [7]. Dengue incidence is also high and has been increasing considerably in Malaysia since 2000 [16]. Starting in 2012, Kudat was the focus of an extensive, interdisciplinary research project aiming to identify the social and ecological drivers of *P. knowlesi* emergence [77]. As part of this project, a 2 × 3 km grid (Fig. 1) encompassing a range of habitats reflecting different land cover types was selected for detailed study of macaque and mosquito vector ecology. This study was based in two villages situated within this grid: Tuboh (06°764'67"N, 116°769'53"E) and Paradason (06°769'57"N, 116°786'18"E). Tuboh is a small village of approximately 20 houses surrounded by patches of clearing, palm trees, rubber trees and secondary forest. Paradason village is situated 1.5–2 km from Tuboh and is also composed of approximately 20 houses. Palm and rubber fields comprise most of the land surrounding Paradason in addition to a large area of secondary forest.

### Resting collection techniques

Three different methods were used to sample resting mosquitoes. The first was the resting bucket trap (RB) [59] which is made from a 20l black plastic bucket lined with black linen cloth (Additional file 1: Figure S1a). RBs were set by placing them horizontally on the ground, with a black cloth soaked in water inside to increase humidity. Mosquitoes were removed from RB’s using a CDC backpack aspirator (John W. Hock, model 1412). The performance of the RB was contrasted with another recently developed method for passive sampling of resting mosquitoes: the sticky resting bucket (SRB) (Additional file 1: Figure S1b). This trap is a modification of the Sticky Resting Box [70] in which the inner surface is lined with sticky surfaces to trap mosquitoes that land on them. The SRB is an RB with an inner lining made of four A4 acetate sheets coated in DeBello rat glue. This was developed as an improvement to the standard RB because it was hypothesized that the sticky surfaces would increase the catch. Mosquitoes affixed to sticky surfaces were removed by cutting out a small square from the acetate sheet. The same acetate sheet was used throughout the week but replaced when more than 5 mosquitoes had been cut from one sheet or if it had become dusty. Both types of resting traps were set up between 12:00–17:00 h on the first day and were re-set after collections each subsequent morning between 6:00–11:30 h.

RB and SRB collections were made daily in all habitat types except for inside houses because of potential intrusion to residents. Instead, mosquitoes resting inside houses were collected using a CDC backpack aspirator [78–80]. A CDC backpack aspirator was used to aspirate mosquitoes inside houses by moving the nozzle in a steady up and down motion along the walls. As the houses were of differing sizes, the time required for full aspiration varied between 3–10 min. Whilst CDC backpack aspiration is regularly used for mosquito surveillance inside houses, its value for sampling mosquitoes resting in outdoor environments, particularly in wilderness areas away from houses, is unknown. To evaluate this, we also conducted a 2-min timed aspiration of all vegetation/objects within a 2 m radius of each RB trap. The height of aspiration was confined to the reach of the aspirator nozzle, i.e. *c.*2 m from the ground. All surfaces and features of vegetation were searched: plant bases, trunks, axils, dorsal sides of leaves and tree holes. In the forest canopy, RB and CDC backpack aspiration collections were not conducted because the operator could not access the forest canopy with the aspirator and lowering the RB traps from the canopy would cause any mosquitoes resting inside to fly out.

RB and SRB traps were set up in pairs positioned 0.3–1.0 m from each other. Traps were placed facing opposite directions to avoid direct competition, whilst being close enough to be exposed to the same environmental conditions. Pairs were positioned 5–10 m from one another and GPS-marked. Maintaining 5–10 m between each SRB-RB pair was not always achievable when they were placed under small houses. Each RB, SRB and 2 min CDC backpack aspiration were single replicates and were used in each habitat type except inside houses and the forest canopy where only CDC backpack aspiration and SRB were used, respectively. Chicken wire mesh with wide holes of one square inch was fixed to the front of SRBs located under and around houses to prevent any larger animals entering and getting stuck. The order in which traps were checked each morning was selected at random to avoid order effects; with some exceptions made to avoid sampling inside houses early the morning when residents were still sleeping.

### Experimental design

Surveillance of mosquitoes resting in domestic, peridomestic and forest settings was carried out over an 8-week period in 2015, with the first 4 weeks spent in Tuboh and the following 4 weeks in Paradason. Within each village, mosquito surveillance was conducted in 8 different habitat types selected to reflect the range of habitats available to mosquitoes in and around human dwellings, and nearby forest habitats where reservoir hosts are present (Additional file 2: Table S1 and Additional file 3: Figure S2). These habitats also represent a gradient arising from deforestation, including mature secondary forest of approximately 10–15 years-old (inside forest, in the canopy and forest edge), palm and rubber plantations, and human settlements (inside, under, and immediately around houses).

Eight households that were easily accessible by motorbike and who consented to participate were recruited from both Tuboh and Paradason. These were subdivided into one group of four households in the north of each village and one group of four households in the south (totalling four groups of four households). The position of each group acted as a focal point from which the selection of sampling points in other habitat types was based. Specifically, an accessible patch of palm or rubber plantation, and of secondary forest, was selected within approximately 400 m of each group of 4 households. Each house (*n* = 19), palm plantation (*n* = 5), rubber plantation (*n* = 4) and forest patch (*n* = 5) were assigned a code so that RB, SRB and CDC backpack aspiration collections made in the same area could be identified (Additional file 4: Figure S3 and Additional file 5: Figure S4). These were defined as ‘spatial clusters’.

For each village, one group of four houses was sampled on week one and week three of the month and the southerly group on weeks two and four. Four nights of trapping were conducted per week. In some instances, a household sampled in the first week could not participate again, therefore a new house in the nearby area was substituted in its place. A total of 19 different households took part in the study, but in each week of sampling a maximum of four houses were visited.

### Mosquito processing

Mosquitoes collected from traps were transported to the central field laboratory in Pinawantai village (8 km from Tuboh). All specimens were then examined under a stereomicroscope and identified to the genus level using the illustrated keys by Rattanarithikul et al. [81–84]. *Aedes* and *Culex* individuals were identified to the subgenus and species level where possible. The sex and gonotrophic stage (unfed, blood-fed, semi-gravid and gravid) of female mosquitoes was recorded. All samples were stored in 95% ethanol at room temperature in microcentrifuge tubes after morphological identification.

### Blood meal analysis

All females categorised as recently blood-fed, based on the presence of blood visible in the abdomen were subject to blood meal analysis by conducting PCR on their stomach contents, following methods of Kocher et al. [85] and Kent [86]. Primers used were FOR (5'-CCA TCC AAC ATC TCA GCA TGA TGA AA-3') and REV (5'-GCC CCT CAG AAT GAT ATT TGT CCT CA-3') to amplify a 358 bp fragment of the vertebrate cytochrome *b* gene [86].

### Data analysis

Statistical analyses were conducted in R version 3.4.2, with the packages *glmmADMB* and *multcomp*. Analyses were performed for specific taxonomic groups that are associated with disease transmission: (i) *Aedes* mosquitoes (including vectors of dengue, chikungunya and Zika virus: *Ae. albopictus* and *Ae. aegypti*); and (ii) *Culex* mosquitoes (including vectors of JE and filariasis: *Cx. quinquefasciatus*, *Cx. fucocephala* and *Cx. sitiens*). GLMMs with a binomial distribution were used to test whether the probability of detecting a mosquito (presence/absence) varied between habitat and trap types. Here the response variable was binary with 0 indicating mosquitoes were absent, and 1 that they were present (≥ 1 individual) in the trap. Fixed explanatory variables fitted habitat and trap type, with additional random effects for sampling date and spatial cluster.

The significance of variables were tested by backward elimination using likelihood ratio tests. A similar approach was taken to model how the abundance of mosquitoes varied between trap and habitat type. Here, the response variable was the number of mosquitoes caught in a single trapping event, with a negative binomial model used to account for the overdispersion in count data.

## Results

### General trends in resting mosquito abundance

Over 31 nights of sampling, 5748 trapping events were conducted from which 2243 mosquitoes were collected (Table 1, Additional file 2: Table S1). Resting mosquitoes were found in all habitat types, with *Culex* spp. (*n* = 1666) and *Aedes* spp. (*n* = 483) being the most abundant (Table 1). Only a few individuals from other genera were collected (*n* = 94, Table 1). These were *Tripteroides* (*n* = 38), *Armigeres* (*n* = 20), *Uranotaenia* (*n* = 9), *Lutzia* (*n* = 5), *Hodgesia* (*n* = 2), *Anopheles* (*n* = 1), *Toxorhynchites* (*n* = 1) and unidentified specimens (*n* = 18). Both male and female mosquitoes were found in resting collections, with the proportion of females being highest in SRB collections (69.6% of 381 specimens) and lowest in RB (29.6% of 1067) and CDC collections (30.9% out of 795). Of the 483 *Aedes* mosquitoes, only 264 could be morphologically identified to species level. Of these, 90.9% were identified as *Ae. albopictus* (*n* = 240) and 9.1% *Ae. aegypti* (*n* = 24) (Additional file 2: Table S2). The remaining specimens were missing key diagnostic features such as scales which prohibited identification. Assuming the species composition was similar in the sample that could not be morphologically identified, the majority of remaining *Aedes* were likely to be *Ae. albopictus*. The proportion of *Aedes* specimens that could be identified to the species level was highest in SRB (*n* = 140, 81.9%), then RB (*n* = 45, 56.3%) and lowest in CDC backpack aspiration collections (*n* = 79, 34.1%); indicating that aspiration methods were more likely to damage specimens during collection.

**Table 1:** Abundance of nine genera of resting mosquitoes (males and females combined) collected using CDC backpack aspiration (CDC), resting bucket (RB) and sticky resting bucket (SRB) methods over 8-week sampling period in 8 habitat types arising from deforestation

Trap	Genus	Inside house	Under house	Around house	Palm plantation	Rubber plantation	Forest edge	Forest ground level	Forest canopy
RB	*Culex*	×	636	163	52	10	13	94	×
	*Aedes*	×	8	20	0	14	18	20	×
	*Tripteroides*	×	1	1	1	0	2	1	×
	*Armigeres*	×	1	0	0	0	0	2	×
	*Uranotaenia*	×	0	1	1	2	0	1	×
	*Lutzia*	×	0	0	0	0	0	0	×
	*Hodgesia*	×	0	0	0	0	0	0	×
	*Anopheles*	×	0	0	0	0	0	0	×
	*Toxorhynchites*	×	0	0	0	0	0	0	×
	Unknown	×	0	2	1	0	1	1	×
SRB	*Culex*	×	31	69	16	5	9	33	12
	*Aedes*	×	8	6	10	33	67	33	14
	*Tripteroides*	×	7	1	0	2	1	3	2
	*Armigeres*	×	1	0	0	0	0	1	1
	*Uranotaenia*	×	0	0	0	0	1	1	0
	*Lutzia*	×	2	0	0	0	0	2	0
	*Hodgesia*	×	1	0	0	1	0	0	0
	*Anopheles*	×	0	0	0	0	0	0	0
	*Toxorhynchites*	×	0	0	0	0	0	0	1
	Unknown	×	3	0	1	0	1	1	1
CDC	*Culex*	63	336	79	5	12	9	19	×
	*Aedes*	3	22	48	9	31	58	61	×
	*Tripteroides*	0	2	3	0	1	2	8	×
	*Armigeres*	0	3	1	1	0	4	5	×
	*Uranotaenia*	0	0	0	0	2	0	0	×
	*Lutzia*	0	1	0	0	0	0	0	×
	*Hodgesia*	0	0	0	0	0	0	0	×
	*Anopheles*	0	0	0	0	0	0	1	×
	*Toxorhynchites*	0	0	0	0	0	0	0	×
	Unknown	1	1	1	0	3	0	0	×
Total		67	1064	395	97	116	186	287	31

×, no resting collections performed

Only a small proportion (122/1666) of *Culex* mosquitoes were identifiable to the subgenus level; 14.9% of those that were trapped with RB were distinguishable to subgenus, 21.2% for SRB and 6.9% for CDC (Additional file 2: Table S3). Thus, the trapping methods followed a similar trend for enabling *Aedes* species identification and *Culex* subgenus identification, with SRB allowing greatest accuracy, followed by RB and then CDC. Within the group of specimens that could be identified to subgenus, the medically important subgenus *Culex* was highly represented (45.1% of those that could be identified). Species within this subgenus were *Cx. quinquefasciatus* (*n* = 29); *Cx. fuscocephala* (*n* = 3) and *Cx. sitiens* (*n* = 3; Additional file 2: Table S4). Members of the subgenus *Culex* were found in all trapping methods (SRB: *n* = 20; RB: *n* = 22; CDC: *n* = 13) and in most habitat types (underneath houses: *n* = 32; around houses: *n* = 9; rubber plantations: *n* = 6; forest at ground level: *n* = 4, inside houses: *n* = 3; palm plantation: *n* = 1) except for the forest canopy and edge (Additional file 2: Table S3).

Only one anopheline mosquito, *An. umbrosus*, was collected (in the forest interior). Pooling across habitat types, SRB collections sampled mosquitoes of a higher number of genera (*n* = 8) than those made by CDC (*n* = 7) or RB (*n* = 5) (Table 1). As a result of low sample sizes of other mosquito genera, statistical analysis was restricted to the genera *Aedes* and *Culex*. Mosquitoes were analysed at the level of genus, given that species identification was only possible for part of the sample.

### *Aedes* spp.

The probability of collecting an *Aedes* mosquito using each of the three trapping methods was very low (*c.*0.01) and differed with trap type (*Dev* = 58.3, *df* = 2, *P* < 0.001) but not habitat (*Dev* = 13.76, *df* = 7, *P* = 0.056). *Aedes* were most likely to be trapped using CDC, then SRB and least likely with RB (Table 2). The mean abundance of *Aedes* per trap was low (< 0.05 mosquitoes/trap), and varied with trapping method (*Dev* = 43.92, *df* = 2, *P* < 0.001) and habitat (*Dev* = 17.94, *df* = 7, *P* = 0.01). It was not possible to test for interactions between trap and habitat type in the full data set as only 1 trap type was used in two of the habitat types (e.g. CDC backpack aspiration - inside houses; SRB - forest canopy). However, a second round of analysis was conducted on the subset of data where all 3 collection methods were used. Here, the abundance of *Aedes* was significantly influenced by an interaction between trapping method and habitat (*Dev* = 187.10, *df* = 8, *P* < 0.001). The mean abundance of *Aedes* collected in RB and CDC did not vary between habitats (Table 3); however, SRBs placed in forest edge habitats collected significantly more than those placed around houses (*P* = 0.01).

**Table 2:** Probability of encountering a resting *Aedes* mosquito per CDC backpack aspiration (CDC), resting bucket (RB) and sticky resting bucket (SRB) trap predicted by binomial generalised linear mixed models (GLMM)

Trap	Predicted probability of *Aedes* presence	Lower 95% CI	Upper 95% CI	Tukey’s test between means
CDC	0.029	0.016	0.053	RB *vs* CDC, *P* < 0.001
RB	0.009	0.004	0.018	SRB *vs* CDC, *P* < 0.001
SRB	0.017	0.008	0.033	SRB *vs* RB, *P* = 0.01

**Table 3:** Abundance of resting *Aedes* mosquitoes per CDC backpack aspiration (CDC), resting bucket (RB) and sticky resting bucket (SRB) traps predicted by negative binomial generalised linear mixed models (GLMM) for 6 habitat types arising from deforestation

Habitat	CDC (95% CI)	RB (95% CI)	SRB (95% CI)
Around house	0.033 (0.011–0.095)	1.944 × 10^-2^ (7.095 × 10^-3^–5.328 × 10^-2^)	0.006 (0.002–0.021)
Under house	0.017 (0.005–0.059)	9.329 × 10^-3^ (2.010 × 10^-3^–4.145 × 10^-2^)	0.010 (0.002–0.047)
Palm	0.020 (0.002–0.179)	1.880x10^-7^ (5.880 ×10^-108^–6.012 × 10^93^)	0.016 (0.002–0.136)
Rubber	0.051 (0.005–0.521)	2.924 × 10^-2^ (4.478 × 10^-3^–1.910 × 10^-1^)	0.057 (0.008–0.415)
Forest edge	0.022 (0.002–0.212)	1.611 × 10^-2^ (1.092 × 10^-2^–2.375 × 10^-2^)	0.071 (0.011–0.463)
Forest ground	0.026 (0.003–0.253)	1.955 × 10^-2^ (1.413 × 10^-2^–2.704 × 10^-2^)	0.037 (0.005–0.246)

### *Culex* spp.

**Fig. 2:**
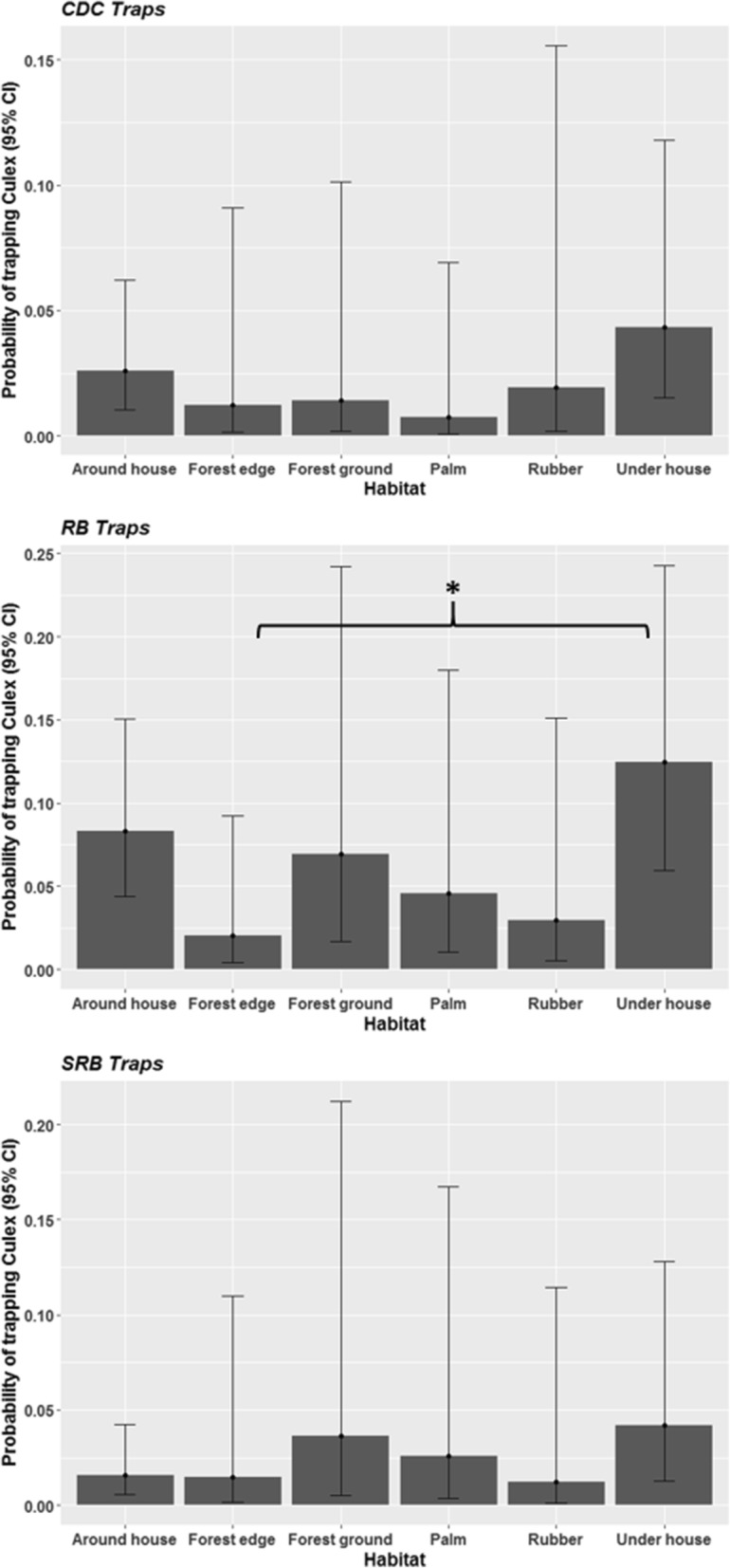
The probability of catching a resting *Culex* mosquito with CDC backpack aspiration (CDC), resting bucket (RB) and sticky resting bucket (SRB) methods predicted by binomial generalised linear mixed models (GLMM). *P < 0.05 (*post-hoc* Tukey’s test)

As with *Aedes*, the probability of collecting a *Culex* mosquito was low on each trapping event (*c.*0.01). Analysis of data collected from all 8 habitat types indicated that the probability of capturing *Culex* differed with trap type (*Dev* = 68.34, *df* = 2, *P* < 0.001) and habitat (*Dev* = 39.58, *df* = 7, *P* < 0.001). Here the probability of sampling a *Culex* mosquito was significantly influenced by an interaction between trapping method and habitat (*Dev* = 175.60, *df* = 8, *P* < 0.001). *Culex* were most likely to be trapped using RB than CDC and SRB (Fig. 2). All three trap types followed the same trend of having the highest probabilities of collecting *Culex* underneath and around houses, and inside the forest, and the lowest in the forest edge and plantations. The probability of sampling *Culex* was similar across all habitats for both CDC and SRB traps. RB positioned underneath homes were more likely to collect *Culex* than those placed at the forest edge (*P* < 0.05).

**Fig. 3:**
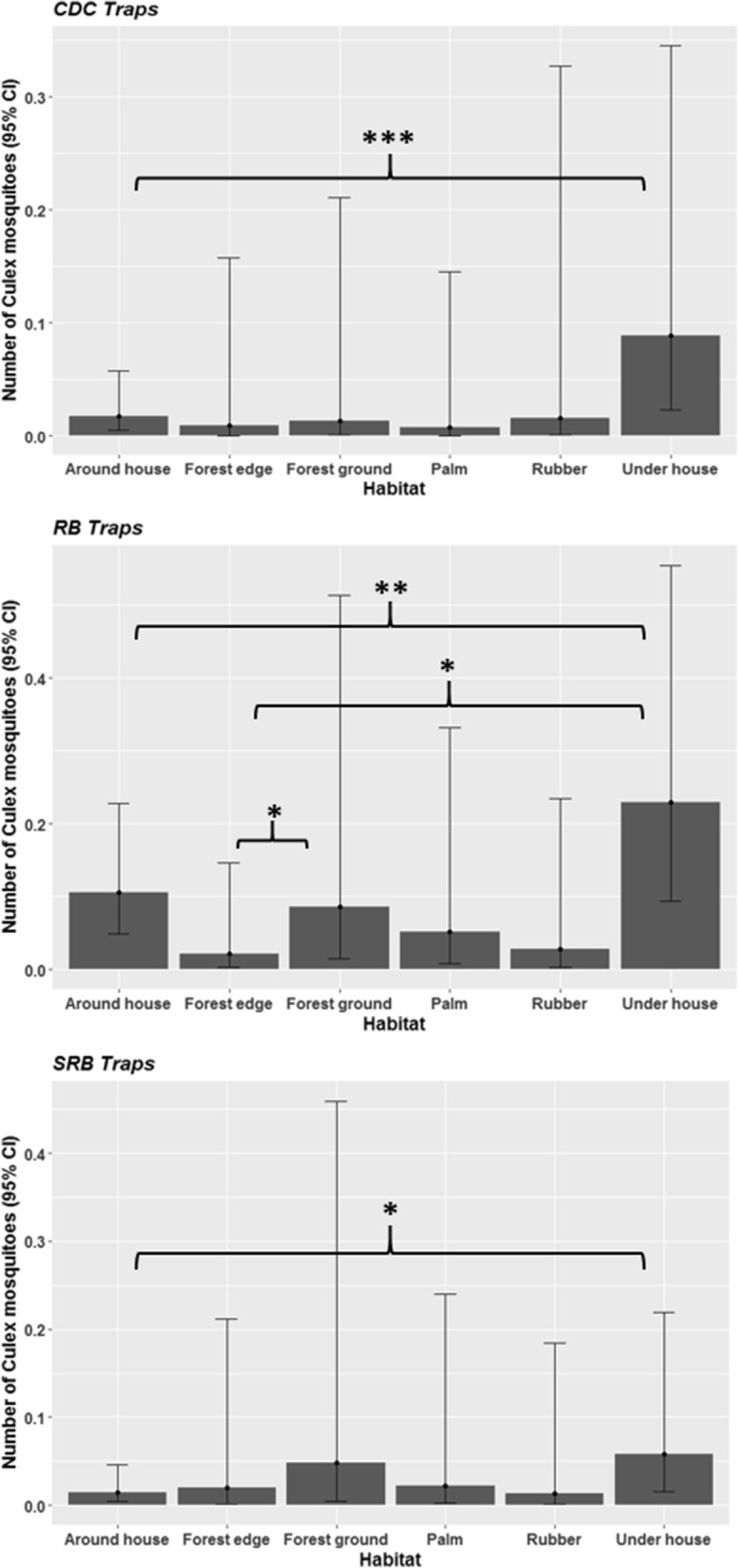
The abundance of resting *Culex* mosquitoes collected using CDC backpack aspiration (CDC), resting bucket (RB) and sticky resting bucket (SRB) methods in six habitat types representing a deforestation gradient. Predicted values obtained with negative binomial generalised linear mixed models (GLMM). *P < 0.05, **P < 0.01, *** *P* < 0.001 (*post-hoc* Tukey’s test)

The abundance of resting *Culex* collected per trap was low (0.1) and differed substantially between habitat (Dev = 60.76, *df* = 7, *P* < 0.001) and trap types (*Dev* = 60.24, *df* = 2, *P* < 0.001). Analysis of the subset consisting of data from habitats in which all 3 traps were tested (6 out of 8 habitats) indicated there was a significant interaction between trapping method and habitat (*Dev* = 246.92, *df* = 8, *P* < 0.001). All three trapping methods followed the same general trend with mean *Culex* abundance being highest in traps placed underneath houses, and lowest in plantations and at the forest edge (Fig. 3). In domestic settings, more *Culex* were found in collections made underneath than around houses with all three trap types (CDC: *P* <0.001; RB: *P* < 0.01; SRB: *P* < 0.05). More *Culex* were collected in RB placed under houses than those at the forest edge (*P* < 0.05). Additionally, more *Culex* were collected from RB placed in the forest interior at ground level than at the edge of the forest (*P* < 0.05).

### Physiological status and blood meal identification

Resting collections are typically used to sample female mosquitoes that have recently blood-fed so that blood meal identification can be performed to confirm host choice. Of the 846 female mosquitoes sampled in this study, 833 were in acceptable condition to assign a feeding status. The majority of these females were unfed (63.3%, *n* = 527/833), with only 15.2% (*n* = 127) appearing to have recently blood-fed. Similar proportions of blood-fed females were obtained with SRB (16.1%, *n* = 43/266), CDC (15.1%, *n* = 38/251) and RB (14.6%, *n* = 46/316) (Additional file 2: Table S5). However SRB traps collected more gravid female mosquitoes (23.3%, *n* = 62/266) than CDC (14.7%, *n* = 37/251) and RB (13.6%, *n* = 43/316). Most blood-fed females (both *Culex* and *Aedes*) were found in collections made under and around houses (Additional file 6: Figure S5 (*Aedes*) and Additional file 7: Figure S6 (*Culex*).

Vertebrate DNA was amplified in only thirty percent of the blood fed mosquitoes that were tested (*n* = 38/127). The majority of these were *Culex* mosquitoes, with most collected around and underneath houses. Blast searches using assembled forward and reverse sequences matched 36 *Culex* with *Gallus gallus* (jungle fowl), 1 *Culex* and 1 *Aedes* (*Stegomyia*) *w*ith human DNA (Additional file 2: Table S6). Blood meals of specimens caught in the forest and plantations did not amplify.

## Discussion

This study represents the first evaluation of two novel methods for sampling mosquitoes resting in a range of domestic, agricultural and forest habitats. Overall these trapping methods had a relatively low probability of detection (*c.*0.1), with mosquitoes being found in < 10% of collections. All resting collection techniques however were successful at trapping mosquitoes in the full range of habitats sampled. *Aedes* and *Culex* mosquitoes were the most abundant and included the known vector species (*Ae. albopictus*, *Cx. quinquefaciatus*, *Cx. fuscocephala* and *Cx. sitiens*). However none of the methods showed promise for collecting malaria vectors, including those responsible for transmitting *P. knowlesi.* Our results provide useful proof-of-principle of the value and limitations of these tools for sampling mosquito vectors and characterizing their resting habitat preferences.

Previous studies had warned of the challenges of collecting outdoor resting blood-fed anophelines in Malaysia [39, 87, 88]. It is interesting to contrast results of the resting catches with those from a 2013–2014 study conducting human landing catches in the same Paradason village, Kudat. The authors reported *Anopheles balabacensis* as comprising the majority of the overall catch and a mean of 7.84 *An. balabacensis* biting man per night [39]. In trapping methods such as HLC, mosquitoes are actively seeking the host thus commonly have greater yields than passive collection methods such as resting collections. Although the sampling efficiency of the resting traps here was quite low, a substantial number of mosquitoes (*n* = 2243) were collected because traps were deployed at high sampling effort (5748 trapping events). Although these trapping methods were unsuccessful for sampling malaria vectors, genera containing other important vector species (*Culex* and *Aedes*) were caught at comparatively high frequency. Members of these genera were widely distributed and found within all habitat types. More *Aedes* were collected in SRBs placed in forest edge habitats than in SRBs placed around houses. Significantly higher abundances of *Culex* were found in collections made under houses than around houses. It is common for the space below houses in Sabah to be utilised by livestock or domestic pets which could explain the higher numbers of mosquitoes resting under houses. Due to the high variability in mosquito catch rates within habitat types, few other clear statistical differences between habitats were detected. A much greater sampling effort and larger sample sizes would likely be required for a robust test of differences between habitats. However, the generally wide distribution of resting mosquitoes across all habitats sampled indicates that there is no single location where most of the resting population could be targeted (e.g. through the spraying of insecticides).

Whilst differences in mosquito abundance between trap types were modest, the three trapping methods compared here did have some differences in efficiency. RB traps and CDC backpack aspiration were more efficient than SRB for sampling *Culex*, whereas more *Aedes* were collected with CDC backpack aspiration and SRB than RB traps. It is unclear why the SRB were not consistently better than the other methods, as we hypothesized the sticky surfaces used in this trap may give it an advantage. In summary, our results indicate that the suitability of specific resting traps differs between mosquito genera, though generally, resting bucket traps and CDC collections caught more mosquitoes than SRB.

One explanation for the differential performance of trapping methods is that they target different sections of the vector population. Here we found that the proportion of gravid mosquitoes (*Aedes* and *Culex*) was higher in SRB than RB or CDC backpack aspiration collections. A previous study in Tanzania also found that the proportion of *Culex* mosquitoes that were gravid was higher in sticky traps than resting buckets (outdoors) and backpack aspiration (indoors) [59]. The authors hypothesized that this may be because the polybutylene-based adhesive mimicked an oviposition odour cue. The glue used in SRBs here was also composed of polybutylenes and polyisobutylenes, and may also have acted as an oviposition cue. The choice of trap therefore likely depends on the target species and required physiological state in certain settings.

All three trapping methods were relatively quick and easy to set up and operate. The SRB involved minimal manual labour to retrieve specimens (as mosquitoes were affixed to a sticky sheet) but required slightly more set-up time for preparation of the glue and acetate. An advantage of the SRB is that they can be left for longer periods of time which is beneficial when placing in difficult to reach habitats such as a forest canopy. RB performed similarly to fixed bursts of two minutes of CDC backpack aspiration in most habitat types. The RB method is more convenient than CDC because only the resting bucket needs to be aspirated instead of a two-minute search by CDC backpack aspiration which is more time-consuming and less standardized.

In making decisions on mosquito trap choice, it is also important to consider the quality of specimens obtained from different methods, and whether they meet requirements for further processing. This study relied on morphological features to identify mosquito species. Scales and hairs are crucial traits for morphological identification to species level. However, we noted that many of these were lost during the trapping process, with a high proportion of *Culex* specimens collected from all three methods being unidentifiable (> 80%). *Aedes* specimens generally remained in better condition, but with notable differences in the proportion that could not be identified between trapping methods. SRB generally kept mosquitoes in a better condition for morphological identification.

The low amplification success of mosquito blood meal hosts was a limitation for the study. A likely explanation could be that the quality of the host DNA was compromised before extraction and amplification. Mosquitoes were examined upon return to the central field station after all resting collections were performed, therefore blood-fed mosquitoes were preserved in 95% ethanol several hours after being collected. There is the possibility that host DNA could have been damaged in this time, thus we recommend to alternatively store immediately in the field upon collection. Previous studies noted that an increase of eight hours after blood meal ingestion significantly reduced the proportion of hosts that could be successfully identified (less than 50% at 15 hours) [89]. Our collections were performed daily, thus exceeding this very short period. As a result, there is a high chance that host DNA in some mosquito blood meals was partially digested in advance of mosquitoes being trapped. Additionally, different habitats may influence blood meal amplification success due to host availability. Around homes there was a notable abundance of blood meal sources e.g. humans, chickens and dogs, therefore mosquitoes collected in those areas would have had the opportunity to feed more recently than mosquitoes collected in areas away from the home such as plantations or forest where there were fewer hosts available. Blood meals of mosquitoes collected further away from the home were more likely to be advanced in digestion which was confirmed with no amplification of blood meals from mosquitoes collected in the plantations and forest. Minor technical issues may have caused low amplification success in our study however mosquito digestion of host DNA within the blood meal is a more prominent concern. Several medically important mosquito vector species were found in this study. This included known vectors of filariasis and Japanese encephalitis [47, 48] (e.g. *Cx. quinquefasciatus*, *Cx. fuscocephala* and *Cx. sitiens*) which are known to be circulating in the study area. These *Culex* species were mainly collected under and around homes, and in palm plantations. In the nearby Ranau District, the most abundant *Culex* species were *Cx. quinquefaciatus* and *Cx. pseudovishnui* [17]. *Culex vishnui*, *Cx. tritaeniorhynchus* and *Cx. gelidus* were also common and all have been incriminated as vectors of JE in Peninsular Malaysia [17]. In Bengkoka Peninsula, neighbouring the Kudat District, *Cx. pseudovishnui*, *Cx. quinquefaciatus* and *Cx. tritaeniorhynchus* are abundant [12, 14]. In Sarawak, Kunjin virus was isolated from *Cx. pseudovishnui* [90] and JE virus was isolated from *Cx. tritaeniorhynchus* and *Cx. gelidus* [91]*.* The variation in *Culex* species between districts may be explained by local ecology and differences in agriculture between regions, e.g. rice fields in Bengkoka.

The majority of *Aedes* mosquitoes that could be identified were *Ae. albopictus*, a suspected vector of dengue virus [46] and also of Zika virus in Singapore [92]. This species was found at highest abundance in forest edge and plantation habitats, possibly due to the availability of both natural shaded breeding sites and artificial containers used for rubber tapping [93]. The increase in availability of domestic breeding habitats such as artificial water containers was previously related to the substantial increase in the abundance of host-seeking *Ae. albopictus* females recorded between the cultivation (1993) and maintenance (1994) stages in an oil palm estate in Sarawak [29]. A further study in Sarawak reported *Ae. albopictus* to be more abundant in agricultural fields (black pepper, cocoa and banana) than in forest sites [46]. Our finding differs from a previous study in Southern Sabah where surveys with oviposition traps found *Ae. albopictus* to be present only near houses, and absent from old growth forest and oil plantations [94]. Similarly, low numbers of host-seeking *Ae. albopictus* were reported in hilly areas covered by primary and secondary forests with alternating areas of scrub and open grass in Bengkoka Peninsula east of Kudat District [14]. *Aedes albopictus* is known to use vegetation for resting [95], and prefer cool, shaded areas for breeding [96]. In combination, this highlights the relatively plastic and exophilic nature of *Ae. albopictus* [48], which allows it to exploit a range of domestic, agricultural and forest settings. Whilst data on sylvatic dengue transmission is not available for this area, it has been reported in other areas of Borneo in patients with a shared history of forest activities (trekking or tree clearance) [46]. More investigation is required to confirm the extent of sylvatic dengue transmission in this area; however, our finding that *Ae. albopictus* is abundant in forested areas flags up its role as a likely vector.

Several potential indications for policy have arisen from this study. One of the most significant implications is that a range of vector species rest underneath houses thus vector control programmes should target these areas with peridomestic insecticide spraying. Secondly, with evidence from human landing catch studies, supporting the presence of anophelines in the study area, we conclude that resting catches are insufficient for examining malaria vector populations. Resting traps should therefore be used as a supplementary tool in conjunction with host-seeking methods. Lastly, important vector species such as *Ae. albopictus* can be found in a range of habitats away from the immediate domestic area. Therefore, efforts to control sylvatic dengue transmission for example would benefit by including habitats away from the home.

## Conclusions

This study demonstrated the new resting buckets and sticky resting buckets can be used to sample a taxonomically diverse range of mosquitoes in a variety of different habitats. However, a limitation of these methods is that they have relatively low sampling efficiency, meaning that they must be deployed at large-scale to generate robust data on mosquito vector resting behaviour and habitat choice. These sampling methods were not successful in trapping malaria vectors but were effective for some *Culex* and *Aedes* mosquitoes. In particular, the sticky resting buckets hold promise for future studies characterising sylvatic dengue transmission. Despite the relatively small numbers of mosquitoes found in these traps, sample sizes were sufficient to indicate that a substantially higher number of *Culex* rest underneath than around homes in this area. Local vector control programmes should consider also targeting these areas with IRS to improve success.

## Additional files

Additional file 1: **Figure S1.** Resting bucket (RB) (**a**) and sticky resting bucket (SRB) (**b**) traps. (TIF 2245 kb)

Additional file 2: **Table S1.** Description of habitat types, number of traps and collections made to investigate mosquito resting behaviour in study area. **Table S2.** Resting *Aedes* mosquitoes collected using CDC, RB and SRB trapping methods in eight habitats arising from deforestation. **Table S3.** Resting *Culex* mosquitoes collected using CDC, RB and SRB trapping methods in eight habitats arising from deforestation. **Table S4.** Resting medically important *Culex* species collected using CDC, RB and SRB trapping methods in eight habitats arising from deforestation. **Table S5.** Blood-fed female resting mosquitoes obtained throughout the study. **Table S6.** Blood meal hosts of engorged female mosquitoes. Hosts were identified using PCR and sequencing of the vertebrate cytochrome *b* mitochondrial gene. (DOCX 48 kb)

Additional file 3: **Figure S2.** Habitats selected to represent a gradient of different microhabitats arising from deforestation. Resting mosquito collections were performed in **a:** inside house; **b**: under house; **c**: around house; **d**: palm plantation; **e**: rubber plantation; **f**: forest edge; **g**: forest interior at ground level; **h**: forest canopy. (TIF 2316 kb)

Additional file 4: **Figure S3.** Tuboh village. Icons indicate sampling areas of different habitat types: yellow pentagons-houses; orange stars-palm plantations; purple squares-rubber plantations; blue triangles-forest patches. Each icon signifies a different sampling area and habitat, and thus was assigned an individual spatial cluster in analysis. (TIF 1228 kb)

Additional file 5: **Figure S4.** Paradason village. Icons indicate sampling areas of different habitat types: yellow pentagons-houses; orange stars-palm plantations; purple squares-rubber plantations; blue triangles-forest patches. Each icon signifies a different sampling area and habitat, thus was assigned an individual spatial cluster in analysis. (TIF 1190 kb)

Additional file 6: **Figure S5.** Physiological status of female *Aedes* collected. (TIF 246 kb)

Additional file 7: **Figure S6.** Physiological status of female *Culex* collected. (TIF 256 kb)

## Abbreviations

CDC: Centre for Disease Control and Prevention backpack aspirator
GLMM: Generalised linear mixed model
JE: Japanese encephalitis
PCR: Polymerase chain reaction
RB: Resting bucket
SRB: Sticky resting bucket
VBD: Vector-borne disease
