# Supplementary figures and images for: Evaluation of resting traps to examine the behaviour and ecology of mosquito vectors in an area of rapidly changing land use in Sabah, Malaysian Borneo
Source: Parasit Vectors. 2018 Jun 14;11:346. doi: 10.1186/s13071-018-2926-1 (PMC6000972; doi:10.1186/s13071-018-2926-1)

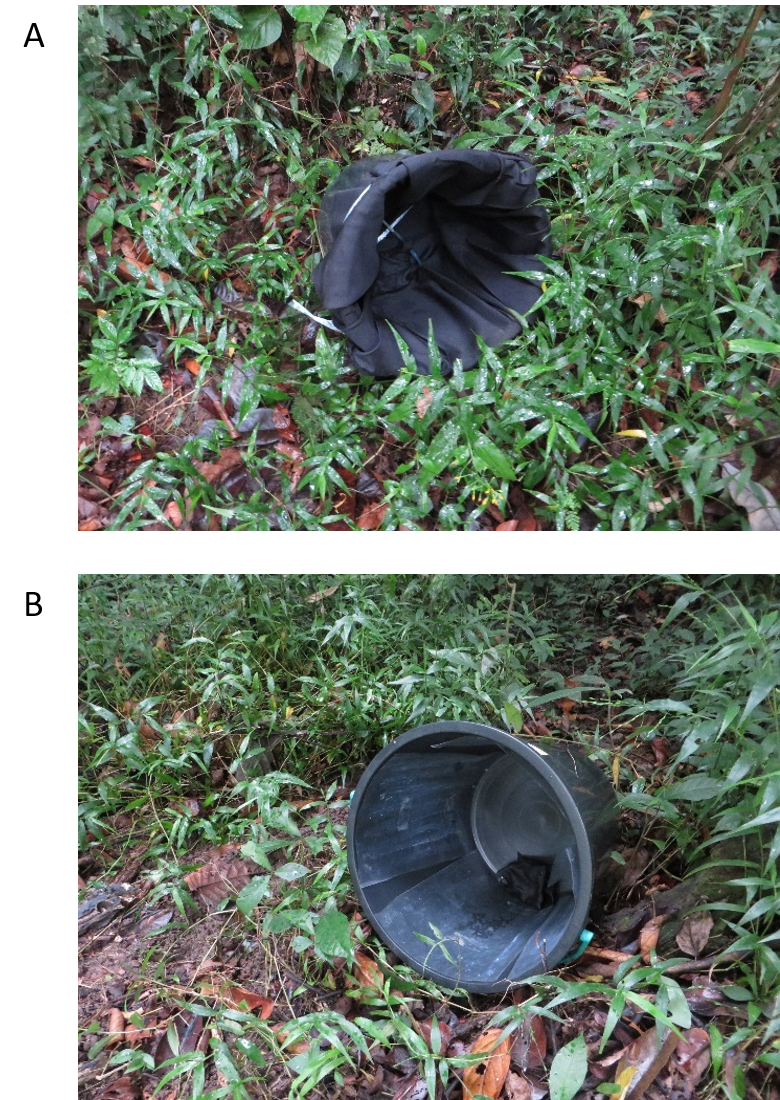

Supplement: Supplementary file 1 — Figure S1. Resting bucket (RB) (a) and sticky resting bucket (SRB) (b) traps. (TIF 2245 kb) [file 13071_2018_2926_MOESM1_ESM.tif]

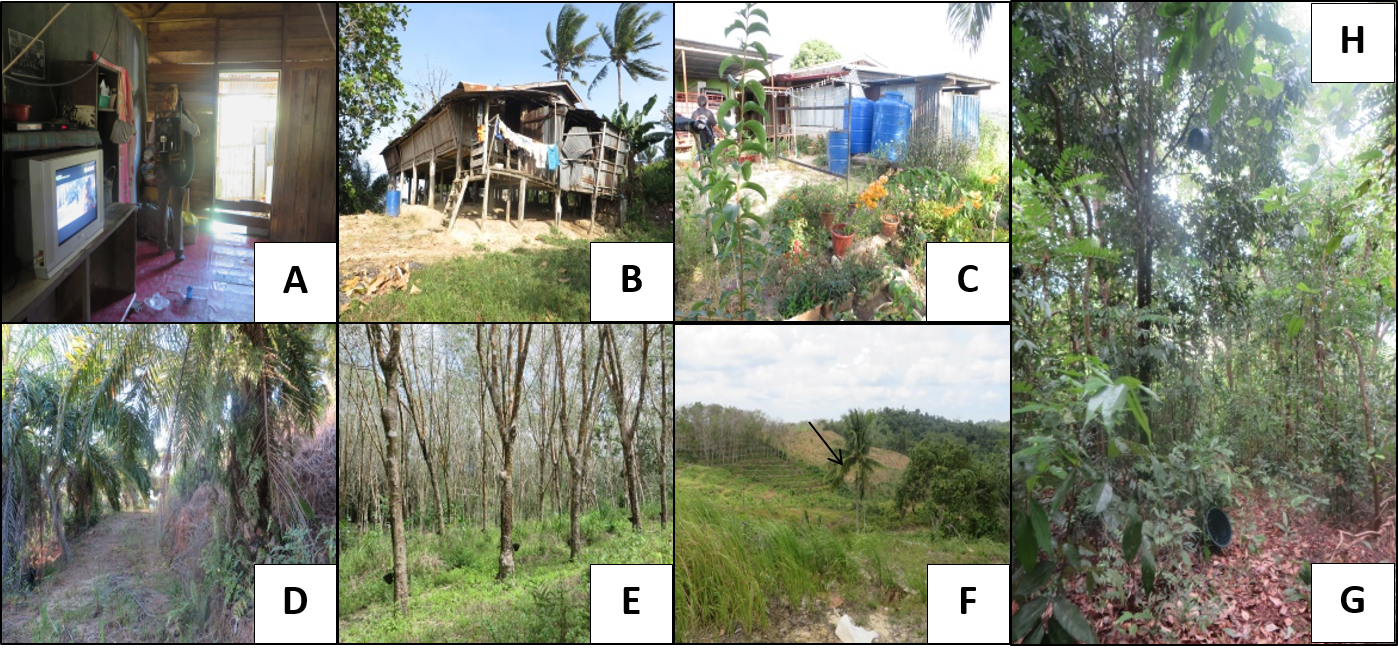

Supplement: Supplementary file 3 — Figure S2. Habitats selected to represent a gradient of different microhabitats arising from deforestation. Resting mosquito collections were performed in a: inside house; b: under house; c: around house; d: palm plantation; e: rubber plantation; f: forest edge; g: forest interior at ground level; h: forest canopy. (TIF 2316 kb) [file 13071_2018_2926_MOESM3_ESM.tif]

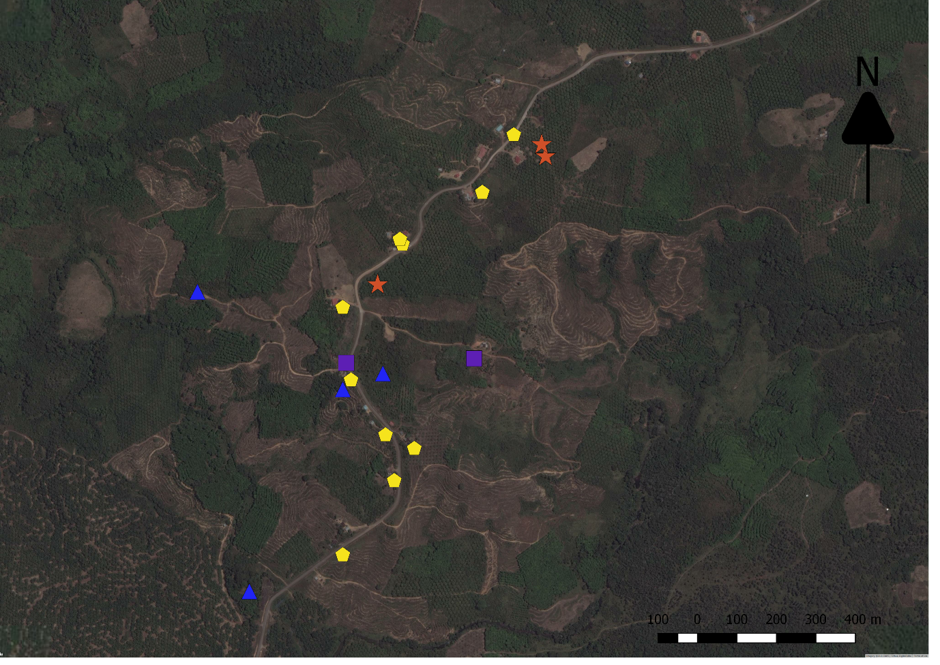

Supplement: Supplementary file 4 — Figure S3. Tuboh village. Icons indicate sampling areas of different habitat types: yellow pentagons-houses; orange stars-palm plantations; purple squares-rubber plantations; blue triangles-forest patches. Each icon signifies a different sampling area and habitat, and thus was assigned an individual spatial cluster in analysis. (TIF 1228 kb) [file 13071_2018_2926_MOESM4_ESM.tif]

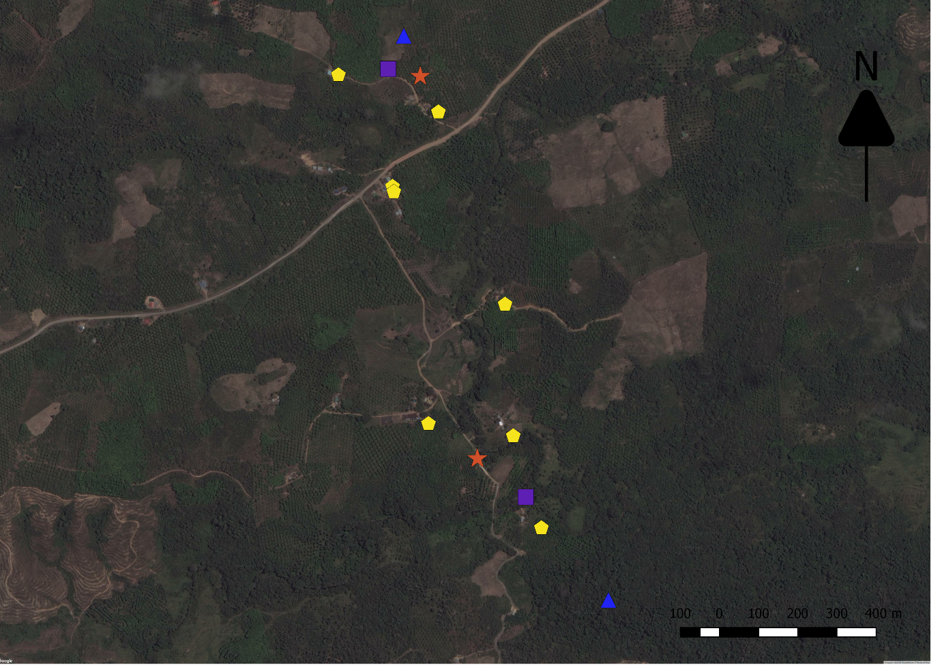

Supplement: Supplementary file 5 — Figure S4. Paradason village. Icons indicate sampling areas of different habitat types: yellow pentagons-houses; orange stars-palm plantations; purple squares-rubber plantations; blue triangles-forest patches. Each icon signifies a different sampling area and habitat, thus was assigned an individual spatial cluster in analysis. (TIF 1190 kb) [file 13071_2018_2926_MOESM5_ESM.tif]

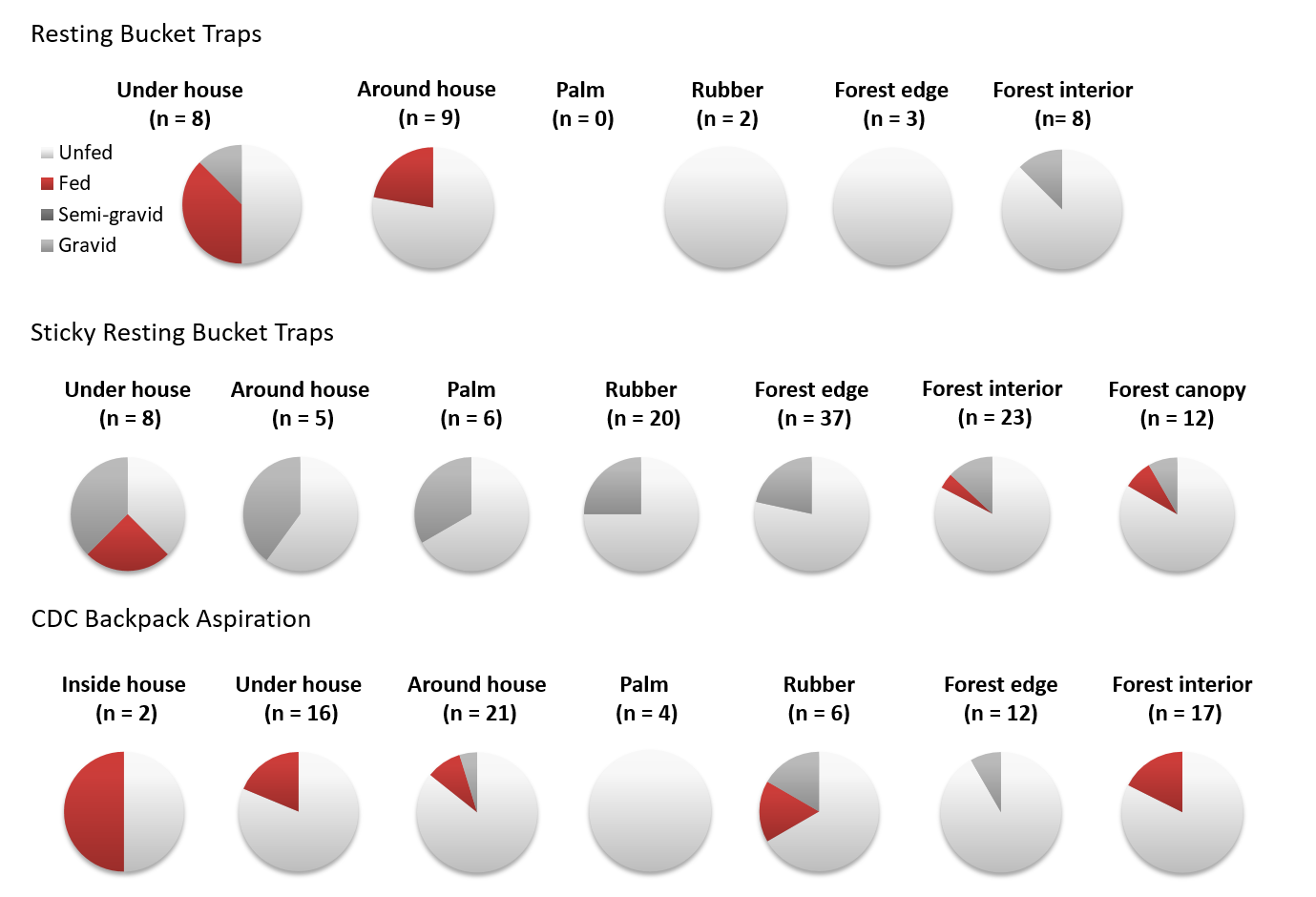

Supplement: Supplementary file 6 — Figure S5. Physiological status of female Aedes collected. (TIF 246 kb) [file 13071_2018_2926_MOESM6_ESM.tif]

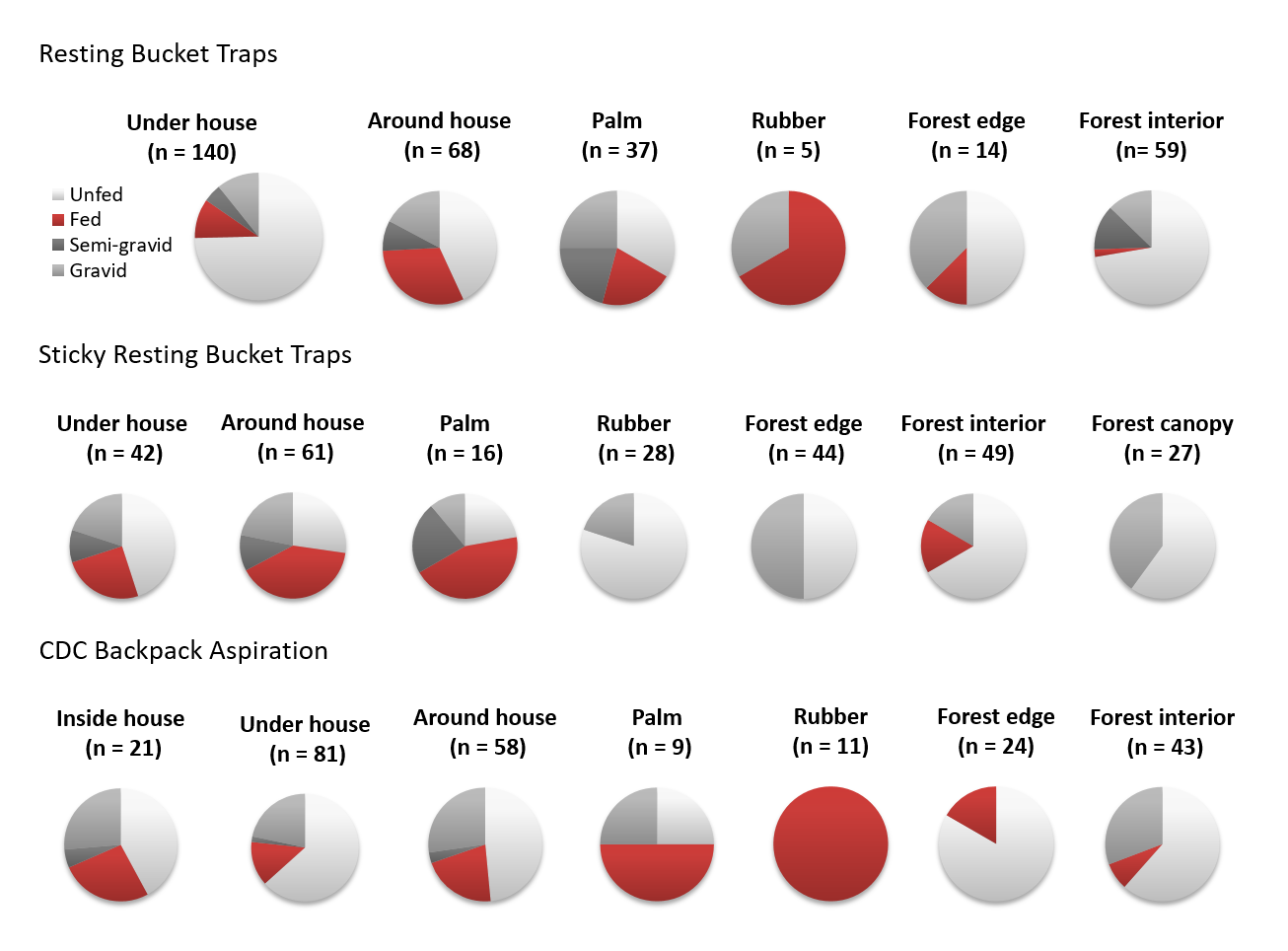

Supplement: Supplementary file 7 — Figure S6. Physiological status of female Culex collected. (TIF 256 kb) [file 13071_2018_2926_MOESM7_ESM.tif]
