# Supplementary material for: Evaluation of resting traps to examine the behaviour and ecology of mosquito vectors in an area of rapidly changing land use in Sabah, Malaysian Borneo
Source: Parasit Vectors. 2018 Jun 14;11:346. doi: 10.1186/s13071-018-2926-1 (PMC6000972; doi:10.1186/s13071-018-2926-1)
Supplement: Supplementary file 2 — Table S1. Description of habitat types, number of traps and collections made to investigate mosquito resting behaviour in study area. Table S2. Resting Aedes mosquitoes collected using CDC, RB and SRB trapping methods in eight habitats arising from deforestation. Table S3. Resting Culex mosquitoes collected using CDC, RB and SRB trapping methods in eight habitats arising from deforestation. Table S4. Resting medically important Culex species collected using CDC, RB and SRB trapping methods in eight habitats arising from deforestation. Table S5. Blood-fed female resting mosquitoes obtained throughout the study. Table S6. Blood meal hosts of engorged female mosquitoes. Hosts were identified using PCR and sequencing of the vertebrate cytochrome b mitochondrial gene. (DOCX 48 kb) [file 13071_2018_2926_MOESM2_ESM.docx]

### Additional file 2

**Table S1.** Description of habitat types, number of traps and collections made to investigate mosquito resting behaviour in study area.

| **Habitat type** | **Description** | **Traps per sampling night** | **Resting collections made per week** |
| --- | --- | --- | --- |
| **Inside house** | All interior walls of every room in the home | 4 x backpack aspiration | 16 x backpack aspiration |
| **Under house** | Houses were raised on stilts ~0.5-1m above the ground, thus collections were performed in the gap between the ground and the house floor | 12 x backpack aspiration  12 x resting buckets  12 x sticky resting buckets | 48 x backpack aspiration  48 x resting buckets  48 x sticky resting buckets |
| **Around house** | The peri-domestic garden area, within 10m of the home | 12 x backpack aspiration  12 x resting buckets  12 x sticky resting buckets | 48 x backpack aspiration  48 x resting buckets  48 x sticky resting buckets |
| **Plantations (palm or rubber)** | Farming areas of 100-200m^2^ where oil palm trees are being cultivated | 12 x backpack aspiration  12 x resting buckets  12 x sticky resting buckets | 48 x backpack aspiration  48 x resting buckets  48 x sticky resting buckets |
| **Forest edge** | The forest fringe at the join between forest patch and area of other land-use | 12 x backpack aspiration  12 x resting buckets  12 x sticky resting buckets | 48 x backpack aspiration  48 x resting buckets  48 x sticky resting buckets |
| **Forest ground level** | 20m inside the forest patch on the forest floor | 12 x backpack aspiration  12 x resting buckets  12 x sticky resting buckets | 48 x backpack aspiration  48 x resting buckets  48 x sticky resting buckets |
| **Forest canopy** | 20m inside the forest hanging in trees at 2.5-9m above ground level | 12 x sticky resting buckets | 48 x sticky resting buckets |

**Table S2.** Resting *Aedes* mosquitoes collected using CDC, RB and SRB trapping methods in eight habitats arising from deforestation.

|  |  | **Habitat type** | | | | | | | |  |
| --- | --- | --- | --- | --- | --- | --- | --- | --- | --- | --- |
| **Trap** | ***Aedes* species** | **Inside house** | **Under house** | **Around house** | **Palm plantation** | **Rubber plantation** | **Forest edge** | **Forest ground level** | **Forest canopy** | **Sum** |
| **RB** | *Ae. albopictus F* | × | 4 | 3 | 0 | 0 | 2 | 3 | × | 12 |
|  | *Ae. albopictus M* | × | 0 | 7 | 0 | 4 | 7 | 6 | × | 24 |
|  | *Ae. aegypti F* | × | 0 | 3 | 0 | 0 | 0 | 1 | × | 4 |
|  | *Ae. aegypti M* | × | 0 | 4 | 0 | 0 | 1 | 0 | × | 5 |
|  | Unknown *Aedes* F | × | 4 | 2 | 0 | 2 | 1 | 4 | × | 13 |
|  | Unknown *Aedes* M | × | 0 | 1 | 0 | 8 | 7 | 6 | × | 22 |
|  | Total | × | 8 | 20 | 0 | 14 | 18 | 20 | × | 80 |
| **SRB** | *Ae. albopictus F* | × | 7 | 3 | 5 | 16 | 27 | 15 | 9 | 82 |
|  | *Ae. albopictus M* | × | 0 | 1 | 4 | 13 | 22 | 7 | 2 | 49 |
|  | *Ae. aegypti F* | × | 1 | 0 | 0 | 0 | 4 | 2 | 1 | 8 |
|  | *Ae. aegypti M* | × | 0 | 0 | 0 | 0 | 1 | 0 | 0 | 1 |
|  | Unknown *Aedes* F | × | 0 | 2 | 1 | 4 | 6 | 6 | 2 | 21 |
|  | Unknown *Aedes* M | × | 0 | 0 | 0 | 0 | 7 | 3 | 0 | 10 |
|  | Total | × | 8 | 6 | 10 | 33 | 67 | 33 | 14 | 171 |
| **CDC** | *Ae. albopictus F* | 0 | 6 | 4 | 3 | 1 | 4 | 5 | × | 23 |
|  | *Ae. albopictus M* | 0 | 2 | 11 | 1 | 3 | 18 | 15 | × | 50 |
|  | *Ae. aegypti F* | 0 | 0 | 1 | 0 | 0 | 3 | 0 | × | 4 |
|  | *Ae. aegypti M* | 0 | 0 | 0 | 0 | 0 | 1 | 1 | × | 2 |
|  | Unknown *Aedes* F | 3 | 10 | 16 | 1 | 5 | 5 | 12 | × | 52 |
|  | Unknown *Aedes* M | 0 | 4 | 16 | 4 | 22 | 27 | 28 | × | 101 |
|  | Total | 3 | 22 | 48 | 9 | 31 | 58 | 61 | × | 232 |
|  | Overall sum | 3 | 38 | 74 | 19 | 78 | 143 | 114 | 14 | 483 |

**Table S3.** Resting *Culex* mosquitoes collected using CDC, RB and SRB trapping methods in eight habitats arising from deforestation.

|  |  | **Habitat type** | | | | | | | |  |
| --- | --- | --- | --- | --- | --- | --- | --- | --- | --- | --- |
| **Trap** | ***Culex* subspecies** | **Inside house** | **Under house** | **Around house** | **Palm plantation** | **Rubber plantation** | **Forest edge** | **Forest ground level** | **Forest canopy** | **Total** |
| **RB** | *Culex* F | × | 5 | 3 | 0 | 0 | 0 | 0 | × | 8 |
|  | *Culex* M | × | 10 | 1 | 0 | 1 | 0 | 2 | × | 14 |
|  | *Culiciomyia* F | × | 0 | 0 | 0 | 0 | 1 | 0 | × | 1 |
|  | *Culiciomyia* M | × | 0 | 0 | 0 | 0 | 2 | 0 | × | 2 |
|  | *Eumelanomyia* F | × | 2 | 1 | 0 | 0 | 1 | 2 | × | 6 |
|  | *Eumelanomyia* M | × | 2 | 1 | 0 | 0 | 0 | 2 | × | 5 |
|  | *Lophoceraomyia* F | × | 2 | 0 | 0 | 0 | 1 | 0 | × | 3 |
|  | *Lophoceraomyia* M | × | 9 | 5 | 0 | 2 | 0 | 0 | × | 16 |
|  | *Oculeomyia* F | × | 0 | 0 | 0 | 0 | 0 | 0 | × | 0 |
|  | *Oculeomyia* M | × | 0 | 0 | 0 | 0 | 0 | 0 | × | 0 |
|  | subgenera unknown F | × | 27 | 46 | 19 | 3 | 5 | 41 | × | 141 |
|  | subgenera unknown M | × | 44 | 72 | 16 | 4 | 3 | 34 | × | 173 |
|  | Total | × | 101 | 129 | 35 | 10 | 13 | 81 | × | 369 |
| **SRB** | *Culex* F | × | 9 | 2 | 1 | 4 | 0 | 1 | 0 | 17 |
|  | *Culex* M | × | 2 | 1 | 0 | 0 | 0 | 0 | 0 | 3 |
|  | *Culiciomyia* F | × | 0 | 1 | 0 | 0 | 1 | 0 | 0 | 2 |
|  | *Culiciomyia* M | × | 0 | 0 | 0 | 0 | 0 | 0 | 0 | 0 |
|  | *Eumelanomyia* F | × | 1 | 0 | 0 | 0 | 1 | 0 | 1 | 3 |
|  | *Eumelanomyia* M | × | 1 | 0 | 0 | 0 | 1 | 1 | 0 | 3 |
|  | *Lophoceraomyia* F | × | 0 | 0 | 0 | 0 | 0 | 0 | 0 | 0 |
|  | *Lophoceraomyia* M | × | 0 | 0 | 0 | 0 | 0 | 1 | 0 | 1 |
|  | *Oculeomyia* F | × | 0 | 1 | 0 | 0 | 0 | 0 | 0 | 1 |
|  | *Oculeomyia* M | × | 0 | 0 | 0 | 0 | 0 | 1 | 0 | 1 |
|  | subgenera unknown F | × | 10 | 36 | 7 | 1 | 4 | 17 | 9 | 84 |
|  | subgenera unknown M | × | 4 | 7 | 4 | 0 | 2 | 12 | 2 | 31 |
|  | Total | × | 27 | 48 | 12 | 5 | 9 | 33 | 12 | 146 |
| **CDC** | *Culex* F | 3 | 3 | 1 | 0 | 1 | 0 | 1 | × | 7 |
|  | *Culex* M | 0 | 3 | 1 | 0 | 0 | 0 | 0 | × | 4 |
|  | *Culiciomyia* F | 0 | 1 | 0 | 0 | 0 | 0 | 0 | × | 1 |
|  | *Culiciomyia* M | 0 | 1 | 0 | 0 | 0 | 0 | 0 | × | 1 |
|  | *Eumelanomyia* F | 0 | 1 | 1 | 0 | 0 | 0 | 1 | × | 3 |
|  | *Eumelanomyia* M | 0 | 1 | 0 | 0 | 0 | 0 | 0 | × | 1 |
|  | *Lophoceraomyia* F | 0 | 6 | 1 | 0 | 0 | 3 | 1 | × | 11 |
|  | *Lophoceraomyia* M | 0 | 4 | 0 | 0 | 1 | 1 | 0 | × | 6 |
|  | *Oculeomyia* F | 0 | 0 | 0 | 0 | 0 | 0 | 0 | × | 0 |
|  | *Oculeomyia* M | 0 | 0 | 0 | 0 | 0 | 0 | 0 | × | 0 |
|  | subgenera unknown F | 16 | 47 | 28 | 4 | 1 | 3 | 10 | × | 109 |
|  | subgenera unknown M | 44 | 269 | 47 | 1 | 9 | 2 | 6 | × | 378 |
|  | Total | 63 | 336 | 79 | 5 | 12 | 9 | 19 | × | 523 |
|  | Overall sum | 63 | 464 | 256 | 52 | 27 | 31 | 133 | 12 | 1038 |

**Table S4.** Resting medically important *Culex* species collected using CDC, RB and SRB trapping methods in eight habitats arising from deforestation.

|  |  | **Habitat type** | | | | | | | |
| --- | --- | --- | --- | --- | --- | --- | --- | --- | --- |
| **Trap** | ***Culex* vectors of medical importance** | **Inside house** | **Under house** | **Around house** | **Palm plantation** | **Rubber plantation** | **Forest edge** | **Forest ground level** | **Forest canopy** |
| **RB** | *Cx. quinquefaciatus* | × | 5 | 1 | 3 | 0 | 0 | 0 | × |
|  | *Cx. fuscocephala* | × | 0 | 0 | 0 | 0 | 0 | 0 | × |
|  | *Cx. sitiens* | × | 0 | 0 | 1 | 0 | 0 | 0 | × |
| **SRB** | *Cx. quinquefaciatus* | × | 0 | 3 | 7 | 0 | 0 | 0 | 0 |
|  | *Cx. fuscocephala* | × | 0 | 3 | 0 | 0 | 0 | 0 | 0 |
|  | *Cx. sitiens* | × | 0 | 0 | 1 | 0 | 0 | 0 | 0 |
| **CDC** | *Cx. quinquefaciatus* | 0 | 1 | 0 | 9 | 0 | 0 | 0 | × |
|  | *Cx. fuscocephala* | 0 | 0 | 0 | 0 | 0 | 0 | 0 | × |
|  | *Cx. sitiens* | 0 | 1 | 0 | 0 | 0 | 0 | 0 | × |

**Table S5.** Blood-fed female resting mosquitoes obtained throughout the study.

|  |  | **Genera of blood-fed females** | | | | | | |  |
| --- | --- | --- | --- | --- | --- | --- | --- | --- | --- |
|  | **Habitat type** | ***Culex*** | ***Aedes*** | ***Uranotaenia*** | ***Armigeres*** | ***Tripteroides*** | ***Lutzia*** | **Unknown** | **Total** |
| **RB** | Under House | 13 | 3 | 0 | 0 | 0 | 0 | 0 | 16 |
|  | Around House | 18 | 2 | 0 | 0 | 0 | 0 | 0 | 20 |
|  | Palm | 5 | 0 | 1 | 0 | 0 | 0 | 0 | 6 |
|  | Rubber | 2 | 0 | 0 | 0 | 0 | 0 | 0 | 2 |
|  | Forest edge | 1 | 0 | 0 | 0 | 0 | 0 | 0 | 1 |
|  | Forest interior | 1 | 0 | 0 | 0 | 0 | 0 | 0 | 1 |
| **SRB** | Under House | 5 | 2 | 0 | 1 | 2 | 1 | 1 | 12 |
|  | Around House | 22 | 0 | 0 | 0 | 0 | 0 | 0 | 22 |
|  | Palm | 4 | 0 | 0 | 0 | 0 | 0 | 0 | 4 |
|  | Rubber | 0 | 0 | 0 | 0 | 0 | 0 | 0 | 0 |
|  | Forest edge | 0 | 0 | 0 | 0 | 0 | 0 | 0 | 0 |
|  | Forest interior | 3 | 1 | 0 | 0 | 0 | 0 | 0 | 4 |
|  | Forest canopy | 0 | 1 | 0 | 0 | 0 | 0 | 0 | 1 |
| **CDC** | Inside House | 5 | 1 | 0 | 0 | 0 | 0 | 0 | 6 |
|  | Under House | 8 | 3 | 0 | 0 | 0 | 0 | 0 | 11 |
|  | Around House | 7 | 2 | 0 | 0 | 1 | 0 | 0 | 10 |
|  | Palm | 2 | 0 | 0 | 0 | 0 | 0 | 0 | 2 |
|  | Rubber | 2 | 1 | 0 | 0 | 0 | 0 | 0 | 3 |
|  | Forest edge | 1 | 0 | 0 | 0 | 0 | 0 | 0 | 1 |
|  | Forest interior | 1 | 3 | 0 | 0 | 1 | 0 | 0 | 5 |
|  | Overall sum | 100 | 19 | 1 | 1 | 4 | 1 | 1 | 127 |

**Table S6.** Blood meal hosts of engorged female mosquitoes. Hosts were identified using PCR and sequencing of the vertebrate cytochrome *b* mitochondrial gene.

| **Genera** | **Subgenera or species** | **Habitat** | **Trap** | **Blood-meal host** | **Number of mosquitoes** |
| --- | --- | --- | --- | --- | --- |
| *Culex* | Unknown | Around house | CDC | *Gallus gallus* | 4 |
| *Culex* | *Culex* (1), *Cx. quinquefaciatus* (1) | Around house | RB | *Gallus gallus* | 10 |
| *Culex* | Unknown | Around house | SRB | *Gallus gallus* | 9 |
| *Culex* | *Culex* (2), *Oculeomyia* (1) | Under house | CDC | *Gallus gallus* | 3 |
| *Culex* | Unknown | Under house | RB | *Gallus gallus* | 2 |
| *Culex* | *Cx. quiquefaciatus* (2) | Under house | SRB | *Gallus gallus* | 2 |
| *Armigeres* | *Arm. moultoni* | Under house | SRB | *Gallus gallus* | 1 |
| *Lutzia* | *Lt. vorax* | Under house | SRB | *Gallus gallus* | 1 |
| *Culex* | Unknown (1), *Cx. fuscocephala* (1) | House indoor | CDC | *Homo sapiens, Gallus gallus* | 2 |
| *Culex* | Unknown | Palm | RB | *Gallus gallus* | 1 |
| *Culex* | Unknown | Palm | SRB | *Gallus gallus* | 2 |
| *Aedes* | *Stegomyia* | Rubber | CDC | *Homo sapiens* | 1 |
